# Supplementary material for: Interleukin-1β Induced Matrix Metalloproteinase Expression in Human Periodontal Ligament-Derived Mesenchymal Stromal Cells under In Vitro Simulated Static Orthodontic Forces
Source: Int J Mol Sci. 2021 Jan 20;22(3):1027. doi: 10.3390/ijms22031027 (PMC7864333; doi:10.3390/ijms22031027)
Supplement: Supplementary file 1 [file ijms-22-01027-s001.pdf]

International Journal of *Molecular Sciences*

MDPI

Supplementary Material

Article

Interleukin-1β induced matrix metalloproteinase expression in human periodontal ligament-derived mesenchymal stromal cells under in vitro simulated static orthodontic forces

Christian Behm, Michael Nemec, Alice Blufstein, Maria Schubert, Xiaohui Rausch-Fan, Oleh Andrukhov and Erwin Jonke

Supplementary Table S1

	**MSC marker** (%)	**Hematopoietic Marker (%)**
**CD29**	97.66 ± 0.19	-
**CD73**	96.29 ± 0.22	-
**CD90**	97.85 ± 0.31	-
**CD105**	96.96 ± 0.42	-
**CD146**	62.63 ± 4.74	-
**CD31**	-	0.46 ± 0.02
**CD34**	-	0.50 ± 0.15
**CD45**	-	2.49 ± 0.26

Supplementary Table S1. Flow cytometry analysis of MSCs' and hematopoietic surface marker expression in hPDL-MSCs. The table shows the percentage of positive hPDL-MSCs for each surface marker. The data are presented as mean ± standard error of the mean.

Publisher's Note: MDPI stays neutral with regard to jurisdictional claims in published maps and institutional affiliations.

© 2021 by the author. Licensee MDPI, Basel, Switzerland. This article is an open access article distributed under the terms and conditions of the Creative Commons Attribution (CC BY) license (http://creativecommons.org/licenses/by/4.0/).
